# Exploring Occupation‐Based Interventions for Sleep Within OTPF: A Systematic Review

**DOI:** 10.1155/oti/5253635

**Published:** 2026-02-25

**Authors:** Ji-Yea Kim, Si-An Lee, Seong-A Lee, Jin-Hyuck Park

**Affiliations:** ^1^ Department of ICT Convergence, The Graduate School, Soonchunhyang University, Asan, Republic of Korea, sch.ac.kr; ^2^ Department of Occupational Therapy, College of Medical Science, Soonchunhyang University, Asan, Republic of Korea, sch.ac.kr

## Abstract

**Introduction:**

Sleep is a critical occupation that affects health and daily functioning, yet occupation‐based approaches to addressing sleep have not been clearly defined within occupational therapy. To clarify current practice, this systematic review examined existing literature and classified occupation‐based interventions (OBIs) for sleep according to the Occupational Therapy Practice Framework (OTPF).

**Methods:**

Searches were conducted in PubMed, Web of Science, and CINAHL for articles published from January 1, 2013, to December 31, 2023, with an updated search performed up to October 27, 2024. Studies were included if they examined OBIs for sleep delivered by occupational therapists, incorporated occupation‐based approaches or theoretical frameworks, and reported outcomes related to sleep quality, sleep participation, or occupational performance. Studies of any design were considered, while review articles, dissertations, conference proceedings, and non‐English or inaccessible publications were excluded. Results were synthesized by classifying OBIs for sleep according to the OTPF.

**Results:**

A total of 7510 studies were identified. After removing duplicates, 6729 studies were screened by title and abstract, and 99 articles underwent full‐text review. Ten studies met all inclusion criteria. The included studies involved diverse populations, regardless of the presence or absence of sleep‐related problems. OTPF‐based classification showed that most interventions primarily focused on sleep preparation, suggesting that current occupational therapy approaches may not fully address broader sleep‐related needs. A multidisciplinary approach may therefore be necessary to address the complexity of sleep problems.

**Conclusion:**

These findings highlight the value of OBIs as theoretically grounded and clinically relevant strategies, while underscoring the need for further development of interventions that integrate both sleep preparation and participation. However, evidence was limited by heterogeneous outcome measures, which precluded meta‐analysis, and by the exclusive focus on occupational therapy interventions, which may not fully reflect the multidisciplinary approaches to sleep.

## 1. Introduction

The occupation of sleep is essential as it has a major impact on people′s health, well‐being, and quality of life [1]. Moreover, sleep deprivation has been shown to negatively affect a wide range of health outcomes, including cardiovascular disease and neurological disorders [2]. It can also reduce motivation to participate in daily activities, ultimately diminishing overall quality of life [3]. Sleep further serves as a foundational occupation that supports engagement in other occupations [4], and sleep‐related problems can interfere with performance in other areas of occupation [5]. Taken together, these findings highlight the need to address sleep problems within the scope of occupational therapy.

This need is also reflected in the Occupational Therapy Practice Framework–Fourth Edition (OTPF‐4), which provides the foundation for defining sleep within the scope of occupational therapy. OTPF‐4 identifies rest and sleep as a distinct area of occupation and further specifies its components through examples. Rest and sleep are defined as restorative activities that support healthy and active engagement in other occupations. Sleep is further categorized into sleep preparation and sleep participation [6]. As a key document that outlines the theoretical and practical basis of occupational therapy, OTPF‐4 offers guidance for applying occupation‐based intervention (OBI) for sleep through its examples and definitions.

OBI does not have a universally established definition [7–9]. However, it is generally recognized as an intervention that supports health, well‐being, and occupational participation in daily life through occupation [10, 11]. Although occupational therapists acknowledge the importance of applying OBI in treatment, time constraints and limited access to appropriate resources have often led to the use of approaches that are not primarily occupation‐based in intervention [7].

Although OBI is a core concept in occupational therapy, existing studies and reviews on sleep‐related interventions have largely concentrated on describing intervention types and their clinical effects, rather than examining these approaches through an explicitly occupation‐based lens [5, 12, 13]. Prior occupational therapy literature includes studies that implemented sleep interventions, such as the use of assistive devices that were not inherently occupation‐centered [12]. Additionally, the literature contains both conceptual reviews examining the theoretical foundations of sleep within occupational therapy and review articles that summarize various sleep interventions and their reported effects [5, 13]. However, across these bodies of work, the focus has remained primarily on procedural descriptions, theoretical discussions, or outcome summaries, with limited attention to the occupational foundations of the interventions or the degree to which they actively engage individuals in meaningful occupation. Consequently, while sleep interventions within occupational therapy have been reported, the extent to which they can be considered genuinely occupation‐based remains insufficiently addressed in the literature. To date, no systematic reviews have specifically examined OBIs within the context of sleep [5, 13]. Moreover, defining OBI for sleep is challenging [7–9], highlighting the need for a clear framework. Although OBIs in the context of sleep have not yet been examined through classification based on the OTPF, numerous studies have reviewed interventions or outcomes using well‐established frameworks such as the International Classification of Functioning, Disability and Health (ICF) [14–16]. Because frameworks such as the ICF and OTPF serve as standardized references in health and occupational therapy, applying a clear framework to classify interventions is essential for understanding how OBIs for sleep are currently being implemented. Therefore, this systematic review is aimed at classifying OBI for sleep using the OTPF and at examining the existing literature through a systematic review.

## 2. Methods

This systematic review was conducted according to the Preferred Reporting Items for Systematic Reviews and Meta‐Analyses (PRISMA). This review was registered at PROSPERO (ID CRD42024611272). ChatGPT was utilized to assist with grammar review and linguistic refinement in the preparation of this manuscript.

### 2.1. Search Strategy and Study Selection

A literature search was conducted in PubMed, Web of Science, CINAHL, and Embase up to October 27, 2024. The search was limited to articles published from January 1, 2013, to December 31, 2023. The following keywords were used: Occupation‐based interventions OR Occupations OR Occupation‐based OR Occupational therapy AND Sleep OR Sleep deprivation OR Sleep disturbance OR Drowsiness OR Insomnia OR Sleep disorder OR Sleep insufficiency OR Sleep problem OR Sleep complaint OR Sleep quality.

The initial screening for eligibility was independently conducted by two reviewers who evaluated the titles and abstracts of the retrieved studies. Any discrepancies between the two reviewers were resolved through consultation with a third reviewer.

### 2.2. Inclusion and Exclusion Criteria

This systematic review included studies that investigated the effectiveness of OBIs for sleep delivered by occupational therapists across diverse populations. Eligible participants were not limited by the presence or absence of sleep‐related problems, reflecting the broad applicability of occupational therapy in addressing sleep issues. Interventions were defined as those incorporating occupation‐based practice approaches or theoretical frameworks in the design and delivery of sleep‐related programs. Studies were included if they employed a comparator such as waitlist controls or traditional rehabilitation methods. A range of methodological designs was considered appropriate for inclusion, provided that the study was aimed at evaluating outcomes related to sleep quality, sleep participation, or occupational performance following the intervention. No specific restrictions were applied to study design, allowing for a comprehensive synthesis of both experimental and observational evidence in this emerging area of practice. Review articles, dissertations, and conference proceedings were excluded, as were articles not written in English or not freely accessible. Two reviewers independently evaluated each study based on the inclusion and exclusion criteria before reaching a consensus on the final selection of studies.

### 2.3. Quality Assessment of Evidence

The evaluation of individual studies was based on the level of evidence (LOE) assessment system developed by the Oxford Centre for Evidence‐Based Medicine [17] (Table 1). Studies were not excluded based solely on their quality; however, any identified methodological limitations were described narratively to provide context for the confidence in interpreting the study findings.

**Table 1:** Characteristics of included studies.

Author and year	Status of participants	Study design and participants	Country	OBI	Model or theory	Assessments	Effect	LOE
Leland et al. [18]	Community‐living urban older adults	Secondary analysis of RCT data, *N* = 217 (EG 119, CG 98), 74.2 years	United States	Lifestyle redesign: A comprehensive and adaptable OBI led by a licensed occupational therapist, focusing on the development of healthy lifestyle behaviors, including sleep	N/A	No standardized tool (mean, SD)	Effective	Level 2b
Leive et al. [19]	Children with neurodevelopmental disorders with insomnia	Pre‐ and postintervention quasiexperimental study, *N* = 30 (EG 22, CG 8), included 3–10 years (no avg. reported)	Argentina	PASIOT (Program to Support Child Sleep from the Occupational Therapy Perspective): A remote, intensive, and individualized intervention coordinated by two occupational therapists and mediated by parents. Grounded in the core features of sleep as an occupation, the program promoted adaptive and maladaptive strategy training and sleep hygiene practices, all tailored to the unique strengths, challenges, and interests of children with NDDs and their caregivers	N/A	SQ, SHQ, and children CSD	Effective	Level 2b
Ho and Siu [20]	Community‐dwelling adults with insomnia	Quasiexperimental study, *N* = 42 (EG 22, CG 20), 57.07 years	Hong Kong	Occupation‐based sleep program: Group sessions are designed to support information sharing and goal setting to encourage the establishment of sleep hygiene routines and positive lifestyle changes. Individual coaching sessions provide follow‐up on action plans, aiming to enhance engagement in meaningful activities and promote occupational balance	PEOP model, theory of occupational balance	C‐ISI, C‐PSQI, activity wristband, OB‐Quest, PHQ9, and GAD7	Effective	Level 2b
Faulkner et al. [21]	People with schizophrenia spectrum disorders	Single‐group mixed‐methods study, *N* = 10, 50.4 years	United Kingdom	L‐DART (Light‐Dark and Activity Rhythm Therapy): Integrates components of CBT‐I with strategies to adjust daytime routines and the home environment, with a focus on enhancing the consistency and rhythm of environmental and behavioral cues (zeitgebers), particularly exposure to light	N/A	ISI, PROMIS‐SD 8a, PROMIS‐SRI 8a, WEMWBS, EQ 5D‐5L, PROMIS‐AP 8a, and CGI‐SCH	Effective	Level 4
Eakman et al. [22]	Military veterans with sleep disturbance	Single‐arm pre–posttest pilot study, *N* = 8, 35.6 years	United States	REST (restoring effective sleep tranquility): A multicomponent CBT‐I intervention led by occupational therapists, designed for military veterans in higher education with service‐related injuries, emphasizing stimulus control and sleep restriction techniques	N/A	Sleep Problems Index II of MOS‐Sleep, PROMIS‐SD, PSQI‐A, DBAS‐10, PROMIS‐AP, PROMIS‐SP, PROMIS‐PI, and performance and satisfaction sections of the COPM	Effective	Level 4
Eakman et al. [23]	Veterans with chronic insomnia	Waitlist‐controlled trial with 3‐month follow‐up, *N* = 15 (EG 7, CG 8)	United States	REST: See Eakman et al. [23]	N/A	PROMIS‐SD, DBAS‐10 scale, PSQI‐A, PSS, PHQ‐Depression scale, GAD, six‐item PTSD checklist, PROMIS‐PI, EMAS, MAAS, PROMIS‐SP, and PROMIS‐AP	Effective	Level 2b
Akbarfahimi et al. [24]	Patients with multiple sclerosis	Pilot RCT, *N* = 20 (EG 10, CG 10), 38.6 years	Iran	Care‐as‐usual + occupational therapy interventions: A combination of common interventions (sleep hygiene education, physical activity, and CBT) and occupational therapy intervention (environmental modification, lifestyle modification)	Occupational balance based on the PEOP model	PSQI, FIS, FSS, SF‐36, and MMSE	Effective	Level 2b
Khanipour et al. [25]	Subjects with hand and upper extremity burns	RCT, *N* = 20 (EG 10, CG 10), 41.8 years	Iran	Occupation‐based intervention: Intervention‐based CO‐OP protocol	CO‐OP	COPM, BAI, SDS, and PSQI	Not effective	Level 1b
Davis‐Cheshire et al. [26]	Adults with sensory sensitivity and insomnia	Multiple‐participant ABA design, *N* = 4, included 21–46 years (no avg. reported)	United States	48 by 72‐in., 12‐pound weighted blanket	N/A	AASP, ISI, CSDM, and ASDQ	Effective	Level 4
Mische et al. [27]	Children with autism spectrum disorder	Single‐subject ABAB reversal design, *N* = 4, 5.5 years	United States	SmartKnitKids Compresso‐T: Compression garment designed to deliver consistent, evenly distributed pressure, supporting sensory regulation by offering deep pressure input to the wearer	N/A	CSHQ, PSI‐SF, Garmin Forerunner 735XT or 935	Not effective (nonstatistical)	Level 4

Abbreviations: AASP, Adolescent/Adult Sensory Profile; ASDQ, Additional Sleep Diary Questions; BAI, Beck Anxiety Inventory; CBT‐I, Cognitive Behavioral Therapy for Insomnia; CGI‐SCH, Clinical Global Impression‐Schizophrenia; C‐ISI, Cantonese Version Insomnia Severity Index; CO‐OP, Cognitive Orientation to daily Occupational Performance; COPM, Canadian Occupational Performance Measure; C‐PSQI, Chinese Version Pittsburgh Sleep Quality Index; CSD, Consensus Sleep Diary; CSDM, Consensus Sleep Diary Morning; CSHQ, Children′s Sleep Habits Questionnaire; DBAS, Dysfunctional Beliefs About Sleep; DBAS‐10, Dysfunctional Beliefs and Attitudes About Sleep Scale–10; EMAS, Engagement in Meaningful Activities Survey; EQ 5D‐5L, 5‐level EQ‐5D version, EuroQol; FIS, Fatigue Impact Scale; FSS, Fatigue Severity Scale; GAD, Generalized Anxiety Disorder Screener; GAD7, General Anxiety Disorder 7; ISI, Insomnia Severity Index; MAAS, Mindfulness Attention Awareness Scale; MMSE, Mini‐Mental State Examination; MOS‐Sleep, Medical Outcomes Study Sleep Measure; NDDs, neurodevelopmental disorders; OB‐QUEST, Occupational Balance Questionnaire; PEOP, person–environment–occupation–performance; PHQ, Patient Health Questionnaire; PHQ9, Personal Health Questionnaire 9; PROMIS‐AP, PROMIS–Ability to Participate in Social Roles and Activities; PROMIS‐PI, PROMIS–Pain Interference; PROMIS‐SD, Patient‐Reported Outcomes Measurement Information System–Sleep Disturbance; PROMIS‐SP, PROMIS–Satisfaction with Participation in Social Roles; PROMIS‐SRI, PROMIS–Sleep‐Related Impairment; PSI‐SF, Parenting Stress Index‐Short Form; PSQI‐A, Pittsburgh Sleep Quality Index Addendum for PTSD; PSS, Perceived Stress Scale; SDS, Self‐Rating Depression Scale; SF‐36, Short‐Form Health Survey; SHQ, Sleep Habits Questionnaire; SQ, sociodemographic questionnaire; WEMWBS, Warwick–Edinburgh Mental Wellbeing Scale.

### 2.4. Data Extraction

Two authors conducted data extraction from the final selection of studies. The extracted features included (1) first author and publication year, (2) status of participants, (3) study design and participants, (4) country, (5) setting, (6) control, (7) OBI, (8) model or theory, (9) assessments, and (10) effect. Any discrepancies among the three reviewers were resolved through discussion.

### 2.5. Data Synthesis

The intervention components described in each included study were reviewed and classified according to the OTPF categories of sleep preparation and sleep participation. Some studies provided only general descriptions of their intervention components, which prevented precise classification under the OTPF categories; these were marked as “not specified.”

## 3. Results

### 3.1. Study Selection

**Figure 1:**
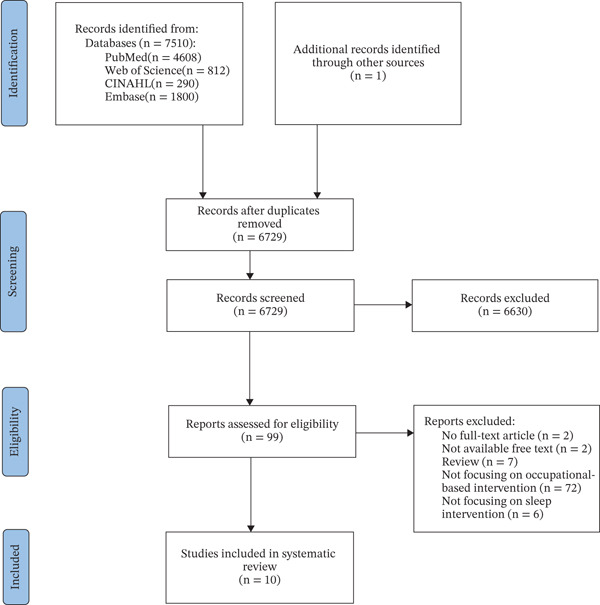
Flowchart of the study selection process.

A total of 7510 studies were identified through our initial database searches. After removing duplicates, 6729 titles and abstracts were screened by two independent reviewers for preliminary screening. Ten studies met the inclusion criteria and were finally selected (Figure 1).

### 3.2. Characteristics of Included Studies

Studies were included with participants of varying status, from children to the elderly, and with or without sleep difficulties. Studies were conducted in the United States (*n* = 5) [18, 22, 23, 26, 27], Argentina (*n* = 1) [19], Hong Kong (*n* = 1) [20], United Kingdom (*n* = 1) [21], and Iran (*n* = 2) [24, 25], with the United States being the most common country where studies were conducted. Three studies used models or theories: PEOP, CO‐OP, and theory of occupational balance [20, 24, 25]. As a result of the OBIs for sleep, eight studies demonstrated effectiveness [18–24, 26], and two studies showed no effect [25, 27] (Table 1).

### 3.3. Classification of OBIs for Sleep Based on OTPF

The content of OBIs for sleep was reviewed according to the definitions of sleep preparation and sleep participation presented in OTPF. Among the four components of sleep preparation, all but “determining the time of day and length of time desired for sleeping and the time needed to wake” (*n* = 2) were implemented in most of the programs. Various sleep intervention programs included a combination of general education, specific education, and personal reflection. General education refers to instruction on general concepts related to sleep, specific education is defined as education on particular sleep‐related guidelines, and personal reflection involves methods aimed at enhancing self‐awareness (Table 2).

**Table 2:** Classification of OBIs for sleep based on OTPF: Sleep preparation.

Program	Sleep preparation			
	Engaging in routines that prepare the self for a comfortable rest, such as grooming and undressing, reading or listening to music, saying goodnight to others, and engaging in meditation or prayers	Determining the time of day and length of time desired for sleeping and the time needed to wake	Establishing sleep patterns that support growth and health (patterns are often personally and culturally determined)	Preparing the physical environment for periods of sleep, such as making the bed or space on which to sleep, ensuring warmth or coolness and protection, setting an alarm clock, securing the home (e.g., by locking doors or closing windows or curtains), setting up sleep‐supporting equipment (e.g., CPAP machine), and turning off electronics and lights
Lifestyle redesign [18]	Participation in activities		General education, discussion, personal reflection	
PASIOT [19]	Participation in activities, breaks		Organization in terms of time and space, sleep routines and rituals	
Occupation‐based sleep program [20]	Relaxation activities		General education, discussion, personal reflection, specific education, application	General education, discussion
L‐DART [21]	Exercise, participation in activities	Setting a sleep schedule	General education, personal reflection, application, review	Home modification (light), using lightbox
REST [22, 23]	Exercise, health management, relaxation activity		General education, personal reflection, specific education (CBT‐I), application	Home modification (light, temperature, noise)
Occupational therapy interventions [24]	Exercise, health management, relaxation activities, specific education (CBT‐I)	Setting a daily schedule, specific education (CBT‐I)	General education, discussion, specific education (CBT‐I)	Home modification (light, temperature, noise, bedding, technology use), specific education (ADL, CBT‐I)
Occupation‐based intervention [25]	Not specified			
48 by 72‐in., 12‐pound weighted blanket [26]				Using weighted blanket
SmartKnitKids Compresso‐T [27]				Wearing compression garment

The L‐DART program and the occupational therapy intervention program included intervention contents for all components of sleep preparation [21, 24]. In contrast, studies that conducted OBIs for sleep using weighted blankets or compression garments were limited to components related to preparing the physical environment [26, 27]. All other programs were included under “engaging in routines” and “establishing sleep patterns,” and two of the programs also addressed “preparing the physical environment” (Table 2).

Sleep participation has three components. Four programs included intervention content related to taking care of personal needs for sleep [20–24], whereas the two components had no corresponding intervention content (Table 3).

**Table 3:** Classification of OBIs for sleep based on OTPF: Sleep participation.

Program	Sleep participation		
	Taking care of personal needs for sleep, such as ceasing activities to ensure onset of sleep, napping, and dreaming	Sustaining a sleep state without disruption	Meeting nighttime toileting and hydration needs, including negotiating the needs of and interacting with others (e.g., children, partner) within the social environment, such as providing nighttime caregiving (e.g., breastfeeding) and monitoring comfort and safety of others who are sleeping
Lifestyle redesign [18]			
PASIOT [19]			
Occupation‐based sleep program [20]	Introduce sleep aids		
L‐DART [21]	Hypnotics, smoking, and caffeine control		
REST [22, 23]	No naps		
Occupational therapy interventions [24]	Alcohol, smoking, and caffeine control, specific education (CBT‐I)		
Occupation‐based intervention [25]	Not specified		
48 by 72 in., 12‐pound weighted blanket [26]			
SmartKnitKids Compresso‐T [27]			

## 4. Discussion

In this systematic review, we classified OBIs for sleep according to OTPF and reviewed the existing literature. Of the 7510 studies reviewed, 10 studies met the inclusion criteria. We categorized the sleep intervention programs used in the included studies based on the OTPF definitions of sleep preparation and sleep participation and found that most interventions primarily focused on sleep preparation.

The participants included in the reviewed studies ranged in age from children to older adults and presented with a variety of physical and mental health conditions. Physical and psychological health problems are closely associated with the quality of sleep [28]. This review also identified that such health‐related issues influenced sleep among participants. Sleep is a vital occupation that plays a fundamental role in maintaining health, and occupational therapy promotes health and well‐being through engagement in meaningful occupations [29]. Given that sleep is a critical occupation across all age groups and health statuses, it should not be confined to a particular population in occupational therapy. Accordingly, the scope of occupational therapy is not limited to specific populations, and this inclusivity equally applies to the sleep domain.

In the categorization of sleep preparation based on the OTPF, components such as general education, specific education, and personal reflection were commonly implemented across various sleep intervention programs. General and specific education was included in five intervention programs, while personal reflection was identified in four. These components closely align with the “education and teaching” model, one of the four core approaches described in the Occupational Therapy Intervention Process Model (OTIPM) [30]. Within this framework, general education, specific education, and personal reflection function as essential tools to enhance client understanding, facilitate behavioral change, and support participation in health‐promoting occupations.

Furthermore, incorporating these strategies contributes to improved sleep hygiene, occupational balance, and overall health promotion. General education provides a foundational understanding of the role of sleep in well‐being [5], while specific education offers concrete and practical techniques for behavioral modification [31]. Personal reflection enhances self‐awareness and supports sustained engagement with therapeutic goals [32], thereby reinforcing the therapeutic value of these educational strategies within OBIs for sleep.

In sleep intervention programs, sleep participation has not been addressed as extensively as sleep preparation. Only a few programs targeted “taking care of personal needs for sleep,” such as reducing smoking, managing caffeine intake, or avoiding daytime naps. Analysis of sleep preparation components revealed that sleep interventions in occupational therapy are typically aimed at resolving sleep problems through lifestyle modifications. These include increasing physical activity, adjusting sleep schedules, and creating environments that support better sleep. These findings are consistent with previous research suggesting that OBIs for sleep seek to minimize the influence of physical dysfunction on sleep, optimize environmental factors, and restructure daily activities with a focus on occupational balance [5]. However, aspects of sleep participation such as “sustaining a sleep state” and “meeting nighttime toileting and hydration needs” were not addressed. These areas are more commonly studied in fields such as sleep medicine and geriatric nursing [33, 34], as they are often associated with physiological decline and chronic health conditions, making them primarily subjects of medical intervention [35]. This underscores the necessity of a multidisciplinary approach to effectively address sleep‐related issues.

Two studies included in this review structured their intervention programs based on occupational balance theory and the person–environment–occupation–performance (PEOP) model [20, 24]. In the study by Ho and Siu [20], the PEOP model was applied to the design of a sleep intervention program under the premise that insomnia may result from the complex interaction of personal, environmental, and occupational factors. In the personal domain, psychoeducation was used to enhance awareness of physiological and psychological factors related to sleep, as well as sleep hygiene practices. In the environmental domain, support was provided for modifying the sleep environment and utilizing assistive devices. In the occupational domain, coaching strategies such as self‐exploration, action planning, and self‐monitoring were used to promote occupational participation and occupational balance. Similarly, in the study by Akbarfahimi et al. [24], a reorganized daily activity schedule was provided, accompanied by environmental modifications such as adjustments to noise, light, and temperature in the bedroom.

The study that applied the CO‐OP model structured its sessions based on the CO‐OP treatment protocol [25]. In the preparatory phase, a collaborative relationship was established with caregivers, and the participant′s daily activities were analyzed to set intervention goals. During the intervention sessions, the global cognitive strategy “goal–plan–do–check” and domain‐specific strategies were employed to facilitate occupational performance. Caregivers actively participated in the process to support the generalization and continued application of the strategies. In the final consolidation phase, Canadian Occupational Performance Measure (COPM) and Performance Quality Rating Scale (PQRS) were used as reevaluation tools to assess changes in occupational performance and to promote the maintenance and transfer of cognitive strategies.

Sleep interventions that incorporated theoretical models and frameworks were distinguished from other programs by their evidence‐based foundation. Notably, these interventions addressed sleep preparation in a more structured and comprehensive manner and extended their scope to include elements of sleep participation—an area that has been relatively underexplored in previous studies.

This study holds the following significance. First, it is the first systematic review to focus specifically on OBI in the context of sleep. By moving beyond outcome‐focused descriptions, this review addresses a critical gap by systematically examining how OBI for sleep aligns with the sleep and rest activities outlined in the OTPF. Specifically, existing interventions were analyzed through the lens of sleep preparation and sleep participation, enabling a structured evaluation of the extent to which these interventions were genuinely occupation‐based. The findings revealed that most interventions emphasized preparatory aspects of sleep, such as routines, environmental modifications, and education, whereas fewer interventions directly addressed sleep participation as an active occupational process. Second, by applying this OTPF‐based classification, the study examined both the theoretical validity and practical implementation of such interventions. Third, it highlighted the importance of general education, specific education, and personal reflection as key components of sleep intervention programs, reflecting the predominant focus on sleep preparation. Lastly, it identified the relative lack of attention given to sleep participation compared to sleep preparation in existing studies and explored potential reasons for this gap.

However, this study has several limitations. First, because the included studies assessed intervention effects using a variety of tools and outcome measures, this review did not conduct a meta‐analysis to statistically synthesize the magnitude of the effects. Second, the analysis was limited to occupational therapy interventions, which may not fully capture the importance of multidisciplinary approaches that are often necessary for effectively addressing sleep‐related issues in clinical settings.

## 5. Conclusions

This review identified that most OBIs for sleep were concentrated on sleep preparation, as categorized by OTPF. Considering the limited focus on other components of sleep, particularly sleep participation, future approaches should incorporate multidisciplinary strategies to comprehensively address sleep‐related concerns. These results emphasize the importance of further advancing OBIs to encompass both sleep preparation and participation, ensuring that interventions are both theoretically comprehensive and clinically applicable.

## Author Contributions

Ji‐Yea Kim and Si‐An Lee contributed equally to this work.

## Funding

The study is supported by the National Research Foundation of Korea, 10.13039/501100003725 (2021R1I1A3041487), and Soonchunhyang University, 10.13039/501100002560.

## Conflicts of Interest

The authors declare no conflicts of interest.

## Data Availability

The data that support the findings of this study are available from the corresponding author upon reasonable request.
